# Providers' perceptions of implementing standardized postpartum family planning: a qualitative study of midwives and nurses in Ghana

**DOI:** 10.1016/j.xagr.2025.100506

**Published:** 2025-05-12

**Authors:** Sarita Sonalkar, Ernest Maya, Chris Guure, Arden McAllister, Dzifa Adimle Puplampu, Roseline Dansowaa Doe, Mary Eluned Gaffield, James Kiarie

**Affiliations:** 1 Department of Obstetrics and Gynecology, University of Pennsylvania Perelman School of Medicine (Sonalkar¸ and McAllister), Philadelphia, PA; 2 Department of Population, Family & Reproductive Health (Maya), University of Ghana School of Public Health, Legon-Accra, Ghana; 3 Department of Biostatistics (Guure, and Puplampu), University of Ghana School of Public Health, Legon-Accra Ghana; 4 Department of Global Health and Population, Harvard T.H. Chan School of Public Health, Boston, MA (Guure); 5 Reproductive, Maternal, Newborn, Child, and Adolescent Health (RMNCAH) Healthy Ageing UHC-Life Course Cluster World Health Organization (Doe), Roman-Ridge-Accra, Ghana; 6 Department of Sexual and Reproductive Health and Research (SRH) (Gaffield, and Kiarie), World Health Organization, Geneva, Switzerland

**Keywords:** postpartum family planning, mobile applications, implementation science

## Abstract

**Background:**

The World Health Organization publishes and maintains evidence-based guidance for the delivery of essential family planning services in the postpartum period**.** Despite significant time and effort in the development of these guidelines in the past decades, a significant unmet need for postpartum family planning persists, exposing an implementation gap. Implementation strategies involving education and infrastructure support can improve family planning uptake and mobile health interventions are a feasible adjunct in improvement of health outcomes.

**Objective:**

We sought to understand provider perceptions of feasibility, acceptability, and appropriateness of implementation of the Postpartum Family Planning Package, which involved provider use of the World Health Organization Medical Eligibility for Contraceptive Use Mobile Application with a one-on-one counseling strategy.

**Study Design:**

We implemented the Postpartum Family Planning Package at three hospitals in Greater Accra and Eastern Regions, Ghana. We conducted in-depth interviews with 23 providers who used the Postpartum Family Planning Package for at least three months. Study design, data collection, analysis, and interpretation were guided by the Consolidated Framework for Implementation Research. We interpreted data using thematic analysis.

**Results:**

Providers indicated that use of one-on-one counseling and the mobile application in hospital-based postpartum care delivery was acceptable, feasible, and appropriate. Respondents described the resultant increased perceived trust between patients and providers as well as more effective and efficient information exchange. Endorsement for the use of the World Health Organization Medical Eligibility for Contraceptive Use Mobile Application by the facility leadership motivated providers to use it. The primary barrier to incorporation of one-on-one counseling was lack of privacy on the postnatal wards.

**Conclusion:**

Providers perceived the implementation of the Postpartum Family Planning Package as feasible, acceptable, and appropriate. Creation of private spaces on the postnatal ward for counseling and continued buy-in by the facility leadership would likely enhance the impact of the strategy. Given the widespread use of mobile technology and the continued unmet need for postpartum contraception, we recommend evaluating this strategy in a broad range of settings internationally, including in lower-resource settings.

**AJOG Global Reports at a Glance**

**Why was this study conducted?**

This qualitative study was conducted to understand provider perceptions of feasibility, acceptability, and appropriateness of implementation of the Postpartum Family Planning Package, which included a provider-facing postpartum family planning educational intervention alongside provider use of the World Health Organization Medical Eligibility for Contraceptive Use Mobile Application in a one-on-one counseling strategy on the postnatal ward.

**Key findings**

Providers felt that using the World Health Organization Medical Eligibility for Contraceptive Use Mobile Application for one-on-one counseling was feasible, acceptable, and appropriate. The primary barrier to one-on-one counseling was lack of privacy on the postnatal wards.

**What does this add to what is known?**

This study adds to the literature on provider perspective on use of technology in the postpartum period setting to address the continued unmet need for postpartum contraception.

## Introduction

The World Health Organization (WHO) publishes and maintains evidence-based guidance for the delivery of essential family planning services in the postpartum period.^1^, ^2^, ^3^ Guidance on postpartum family planning service delivery, including which methods are safe in patients with underlying medical conditions or based on timing of delivery, and how these methods should be prescribed or delivered, has been published in a variety of documents.^4^, ^5^, ^6^ Despite significant time and effort in the development of these guidelines in the past decades, a significant unmet need for postpartum family planning persists.^7^^,^^8^ In a systematic review and meta-analysis of postpartum family planning use in low-income countries, the pooled prevalence of postpartum contraceptive use was only 37%, illustrating an implementation gap.^9^

The immediate postpartum period is an important time to address women’s family planning needs, given that they are interacting with the healthcare system, and many contraceptive methods are appropriate immediately after delivery. Women need evidence-based, individually relevant information in order to initiate a method during this time.^10^ Postpartum providers should be equipped to counsel regarding contraception immediately after delivery, equipped with education and tools that fill gaps in their knowledge, and address the issue of time management.^11^

Implementation strategies involving education and infrastructure support can improve family planning uptake.^12^ Mobile health interventions are a feasible adjunct in improvement of health outcomes,^13^ and can be widely disseminated to a broad range of locations and providers. The World Health Organization Postpartum Family Planning Compendium aids providers in making evidence-based postpartum family planning recommendations, and was pilot tested in Accra, Ghana.^3^^,^^14^ The results from our pilot work informed the development of the Medical Eligibility Criteria (MEC) for Contraceptive use Mobile Application (App), a mobile version of the Medical Eligibility for Contraceptive Use, a document that contains eligibility guidance regarding contraceptive methods in patients with medical conditions and characteristics. In addition to a process for screening clients for method medical eligibility as per the existing WHO guidelines, the additional inclusion of a preference section in the app enables providers to guide clients to choose contraceptive methods that meets clients’ particular preferences in terms of bleeding patterns, and duration of use. From October 2020-September 2021, our team conducted a stepped wedge trial of the multifaceted Postpartum Family Planning Package implementation strategy.^15^ Briefly, this strategy combined provider use of the WHO MEC App along with one-on-one counseling on the postpartum ward. Here, we report the findings of our sub-study conducted after the main trial, in which we sought to qualitatively explore providers’ perception of feasibility, acceptability, appropriateness and sustainability of using the Postpartum Family Planning Package in the postpartum period.

## Materials and methods

We conducted a prospective, qualitative study of family planning providers at three district hospitals in Ghana: Maamobi General Hospital and Ga West Municipal Hospital (Amasaman) in the Greater Accra region, and Nsawam Government Hospital at Nsawam in the Eastern region. These facilities provide general clinical services and have family planning clinics situated within the facilities but separate from the postnatal wards. They serve as primary care facilities as well as referral facilities for health centres and maternity homes within their catchment areas.

We employed qualitative methods to assess provider perceptions of the Postpartum Family Planning Package implementation strategy, which involved promotion of the WHO Medical Eligibility Criteria for Contraceptive use Mobile Application as a tool during one-on-one provider-patient counseling on the postnatal ward. This implementation strategy had been rolled out as a part of a stepped wedge implementation effectiveness study from October 2020 to September 2021. After the implementation study was completed, we conducted in-depth interviews with providers from November to December 2021. We used purposive sampling across three hospitals, and included any health care provider providing postpartum care on the postpartum ward who had used the Medical Eligibility Criteria for Contraceptive use Mobile Application for one-on-one counseling for at least three months. Students were excluded. All providers used their own mobile phones on which they downloaded and stored the mobile app.

Prior to initiation of our implementation strategy, women due for discharge on a given day after childbirth were grouped together and provided with general advice on the importance of family planning and birth spacing for both mothers and their newborns. This was done without the structure of a WHO guidance tool. Women who desired initiation of family planning methods were asked to go to the family planning clinic for counseling and service provision. Our intervention itself was the WHO Medical Eligibility Criteria for Contraceptive use Mobile Application, recommended to be used during one-to-one postpartum counseling on the postpartum ward. The App was used alongside the patient during one-on-one counseling, while the provider inquired about and inputted medical conditions and patient preferences. Recommendations based on the app were used to guide contraception counseling discussions at the bedside. The broader components of the implementation strategy included the following: (1) a one-day educational seminar for postpartum clinical providers on postpartum family planning guidance and counseling, (2) administrative recommendation and support for use of WHO family planning guidance using the Medical Eligibility Criteria for Contraceptive use Mobile Application during clinical care,^16^ and (3) administrative recommendation, support, and normalization for counseling in a one-on-one rather than group format.^15^ Our educational seminar included didactic content related to medical eligibility, patient-centered shared decision-making techniques,^17^^,^^18^ postpartum contraceptive methods, and safety of contraceptive methods in the postpartum period.^4^ In addition, we utilized case-based education and ensured all providers downloaded the mobile application from either Google play store for android phones or the App store for iPhones. Training sessions were facilitated by the investigators and four Ghanaian consultant Obstetrician Gynecologists. The primary outcome of the implementation strategy was provider discussion of all appropriate family planning methods.^15^

### Data collection

Trained research coordinators met with the study participants in person to determine their eligibility. After obtaining consent, they recorded demographic information and conducted approximately 30 to 45 minute in-depth interviews. Research coordinators (including author D.A.P., who had either completed a Masters in Public Health or were MPH students) conducted interviews in English in private, audio recorded in private settings and then transcribed. Research coordinators had previously worked on site for a prior study, and were known to some of the participants. The research team iteratively evaluated the interviews conducted and field notes in order to assess for thematic saturation and determination of the final sample size.^19^, ^20^, ^21^

### Interview guide

Our interdisciplinary team, guided by the Consolidated Framework for Implementation Research (CFIR), developed the in-depth interview guide.^22^ We addressed feasibility, acceptability, and appropriateness of the three elements of the implementation strategy: educational intervention, use of the Medical Eligibility Criteria for Contraceptive use Mobile Application in counseling, and one-on-one counseling. Additionally, for each of the three elements we used constructs within the CFIR to create question prompts that addressed the following: intervention characteristics (including evidence strength and quality, relative advantage, adaptability, design quality and packaging), inner setting (including structural characteristics, networks and communications, culture, and implementation climate), and sustainability (Supplementary Material).

### Analysis

The transcripts were imported into NVivo version 12 (QSR International, Burlington, MA, USA) for analysis. We developed a comprehensive codebook of *a priori* concepts from the CFIR as well as other concepts that arose inductively. We conducted a line-by-line reading of all transcripts, and relevant portions of statements made by respondents were coded onto existing nodes and new nodes. Trained research staff members then double-coded the interviews. The coding team met individually and with the extended research team to discuss and resolve all coding discrepancies by consensus. The investigative team then summarized the codes, analyzed them as a group, and identified themes.

### Ethical consideration

The study was approved by the University of Pennsylvania Institutional Review Board (Protocol ID 834804), the WHO Ethics Review Committee (CPN A65990) and the Ghana Health Service Ethics Review Committee (GHS-ERC: 15/08/19). All study participants provided written informed consent before the interviews.

## Results

We conducted 23 in-depth interviews (IDIs) from November to December 2021; 21 participants were midwives, and 2 were nurses. We conducted 6 interviews among providers at Ga West Municipal Hospital (Amasaman), 8 at Maamobi General Hospital, and 9 at Nsawam Government Hospital. Themes, supportive quotes, and associated implementation constructs including the CFIR constructs used in guiding the analysis are shown in Table. The next section presents the identified themes organized by intervention elements of the Medical Eligibility Criteria for Contraceptive use Mobile Application and one-on-one counseling, along with the implementation constructs that guided the analysis (in parentheses).

**Table:** Themes and Representative Quotes Arising from Qualitative Interviews with Health Care Providers who used the Postpartum Family Planning Package in Ghana, November to December 2021

Implementation construct^22^	Theme	Representative quote
*Acceptability and appropriateness*	*Acceptability of training*	“…I liked the role play they did with us at the end of it but because at the beginning I wasn’t paying much attention, the role play got me more concentrated and then it also helped me to be able to use the app.” (Midwife, Amasaman)
	*Acceptability and appropriateness of app use during clinical care*	"I was supposed to tell the client I will be using the phone for the app but I took the phone and I started, then I realized her face had changed so I told her that madam it’s the same work but it’s on our phones now…so that made me show the first screen to her then I realized she was more comfortable so the subsequent clients that came I tell you about it and they are relaxed." (Midwife, Amasaman)
	*Acceptability of one-on-one counseling*	“…it makes the patient pay attention to you, because you are standing by her and …you are talking to her one on one so there’s attention and you established a rapport before starting so there’s some kind of interest, she will show interest.” (Midwife, Maamobi)
	*Acceptability is increased by learning from the app*	"the part I like is those with medical conditions, because previously you have to wait for your in-charge and discuss with her, this woman has this condition, what can she use? But with this one you just click the condition, and it will give you the methods that she can use." (Midwife, Amasaman)
*Feasibility*	*Privacy is essential to the PPFP strategy success*	“The one-on-one counseling makes the client to have trust because nobody is there, and how you started with her makes her to know that this nurse is good, so even if I tell her everything, there’s nobody here, and it is just the two of us so she will freely tell you anything, there’s some kind of confidentiality.” (Midwife, Maamobi)
		“The one-on-one counseling, though it’s good but sometimes you just don’t get the privacy, our patients are plenty… if we have a corner here for just one on one counseling then you can bring the patient here and get the information. Sometimes you are with them at their bed sides, though you’ve lowered your voice…they will be looking around to see if somebody is listening or something…” (Midwife, Amasaman)
*Relative advantage*	*Time spent using one-on-one counseling*	“This makes the client choose what they want but the large counseling you will talk and talk, and nobody will choose a method. This one [one-on-one counseling] you go to the client, you don’t waste time, you can spend 5 minutes for each client (Amasaman 2)
		…if they come in, they are still in doubt as to which particular method to use but once you tell them this 1,2, 3 is best and with evidence it saves the stress” (Midwife, Amasaman)
		“…it’s a very good thing but then sometimes too it takes time especially when you have to finish with the client and then you have to do another thing on the ward or maybe attend to another client before the person…” (Amasaman 1)
	*Reduced cognitive energy*	“It saves you the stress of having to think which method is best for her, because if she tells you she’s hypertensive you will just input and then it gives you whatever method is good so it’s convenient and easy to use” (Amasaman 3)
		“With the introduction of this app, you know, with the old way of counseling, with the family planning you are expected to know so much like with the various types of contraceptives, advantages, the disadvantages, you have to have everything in your head but with this app you know it’s concise and precise, it’s just been given step by step so you just look at it then, so in a way it makes the counseling kind of easy.” (Midwife, Maamobi)
	*Improved patient comprehension*	“The one-on-one counseling makes the clients to understand what you are saying but the group counseling some will understand but some won’t understand…” (Midwife, Maamobi)
		“The advantage is, [the app] gives much information and serves as a safety guidelines when counseling so you won’t make any mistake or miss or misinform the client, this one has helped us to help the client to do the right method, so when the client asks you a question you won’t fumble and you can adequately help her on that, so this app has helped us a lot, and people are getting the contraceptives.” (Midwife, Maamobi 8)
		“Without the app…you just say it out of mind, we have IUD, we have this and that so when you want it you come, but now that there’s the app, the app will give you knowledge about it, so you tell the client, this is the side effects, if you have this it’s not good for you, this is good so now that we have the app we can give more information to the client for them to really understand.“ (Nurse, Nsawam)
*Culture*	*Managerial support*	“Yes, they do [support use of the app], because when you are educating a client and assuming your superior is passing by, she will ask “you are educating your client err, well done, but make sure you use the app.” (Midwife, Maamobi)
*Sustainability*	*Expansion to the antenatal setting will help the program be sustainable*	“It should start right from antenatal, if the client comes, even though she’s now pregnant, she should be spoke to about family planning… and after delivery you still talk to her then postnatal” (Midwife, Amasaman) “...it doesn’t take a day to change the mentality of someone, it has to be continuous, right from antenatal it’s in the mind of the client and part of our everyday dealings with the pregnant women.” (Midwife, Nsawam)
	Workplace culture affects sustainability	“The hospital has its own goals and objectives and then one of their goals is in line with the Millennium Development Goals is to help reduce maternal death and mortality so if they have it as part of theirs as a hospital, we know family planning also pays a major role in it, so they have embraced it and it’s helping.” (Midwife, Amasaman) “One of the hospital goals is, we are client centered. And I think this app is solely centered on the client and we have the goal where we say we are innovative, and this app I think is an innovative thing that has been introduced, so in a way, it aligns with some of the goals of the facility.” (Midwife, Maamobi)

### Use of the medical eligibility criteria for contraceptive use mobile application during postpartum family planning counseling

#### Use of a mobile application requires prefacing a conversation with clients (appropriateness, acceptability)

Many respondents discussed that using an electronic counseling tool during clinical care should involve a clear explanation of what the mobile phone was to be used for, with patients in case patients thought the providers were inattentive in using their phone for personal means. However, once the use of the mobile application was explained to clients, respondents discussed that counseling often felt simpler, streamlined, and more structured, than the previous model of group counseling. One respondent noticed that clients were more likely to choose methods with this strategy.

#### Use of the app allowed more independent practice (relative advantage compared to prior practice)

Providers also found that the mobile application helped them to practice more independently, eliminating cross-checking of clinical concepts with their supervisors.

### One-on-one postpartum family planning counseling

#### Privacy and confidentiality are essential elements in one-on-one counseling (feasibility)

Ensuring privacy and confidentiality were major themes that emerged throughout the interviews, and related to feasibility, acceptability, appropriateness, intervention characteristics, and context. Providers felt that the structure of one-on-one counseling was more acceptable to patients due to the clients’ perceptions of privacy. The majority of the respondents felt that one-on-one counseling afforded a chance to build rapport and thus more engaged provider-patient interactions. In addition, the one-on-one counseling allowed for individualization of counseling to aid in more tailored discussion of relevant contraceptive methods. One-on-one counseling allowed providers to elucidate the specific areas in which clients needed explanation and allowed understanding of the clients’ medical conditions; this resulted in deeper and more meaningful counseling. However, many discussed that the structure of the labor ward with many other patients close by made it difficult to have private conversations, and at times would be a hindrance to patient disclosure of information.

### Pairing app use with one-on-one counseling

#### Time management considerations in the use of the intervention (feasibility, appropriateness)

A few respondents discussed that time was a barrier to one-on-one counseling especially when client volume was high. However, many respondents felt that using the mobile application actually saved time due to the efficiency of the mobile application and the ability to determine quickly contraceptive choice. In addition, the convenience of the mobile application made counseling faster because providers concentrated only on the recommended methods, and reduced providers’ cognitive energy in remembering the many details of contraceptive method characteristics and eligibility.

#### Improved information exchange (appropriateness)

Respondents felt that clients better understood the information and allowed better information exchange when counseling was done individually. Many respondents stated that the trusted mobile application provided them with guidelines to follow during counseling which increased their confidence in counseling. The rapport built with one-on-one counseling alongside the confidence that providers had in the material they were presenting enhanced the patient-provider relationship.

### Sustainability considerations

#### Managerial support is essential for sustainability (implementation climate)

Participants indicated that sustainability was possible as the leadership embraced the use of the mobile application for the one-on-one counseling, evidenced by leadership’s regular reminders to use the application, their attendance at training sessions, and their granting of protected time to attend training.

#### Sustainability requires periodic training of staff and motivation by champions and supervisors (networks and communications)

Study participants recommended that all staff learn to use the app and employ it at every possible client encounter. To sustain the benefits of the Postpartum Family Planning Package intervention, ongoing or refresher training was described as necessary. Some participants suggested introducing a mechanism to verify that staff are using the app during client encounters to ensure provider accountability. Additional considerations include introducing the mobile application as part of a ‘family planning month’ promotion and introducing its use prior to delivery. Respondents suggested that hospitals could facilitate the use of the mobile application by providing electronic tablets or data credit to remove burden on staff to use their personal devices.

#### Workplace values are related to sustainability (culture)

Respondents stated that workplace culture can support the use of the mobile application: some hospitals highly promote family planning care delivery as part of their values. They also stated that the use of the mobile application for counseling supported the vision and goal of the facility.

## Comment

### Principal findings

In our qualitative study of providers exposed to the Postpartum Family Planning Package, which included promotion of the Medical Eligibility Criteria for Contraceptive use Mobile Application, provider education, and one-on-one counseling on the postnatal ward, we found that the package was overall feasible, acceptable, and appropriate. The package had benefits including increased perceived trust between patients and providers as well as a more effective and efficient exchange of information. The primary barrier to the use of the Postpartum Family Planning Package was lack of privacy on the postnatal wards. The providers suggested incorporation of the package earlier on in care during the antenatal period and creating private spaces on the postpartum ward in order to facilitate personalized discussions about family planning. In general, implementation of the Postpartum Family Planning Package was in alignment with facility mission and values, and was supported by leadership; these factors, along with ongoing training, were thought to contribute significantly to sustainability.

### Results of the study in the context of what is known

Our findings add to the literature that systems change including provider education, offering point of care contraception, and attention to infrastructure can increase quality of contraception care. A recent systematic review of implementation strategies, facilitators, and barriers to scaling up and sustaining post pregnancy family planning showed that attention to infrastructure increased postpartum contraceptive use.^23^ A qualitative study evaluating provider perspectives of a postpartum family planning and postpartum intrauterine contraceptive device training and intervention in Nepal revealed support for the program, but also highlighted infrastructure challenges regarding lack of a dedicated family planning counselor, work overload, lack of private space for counseling, lack of counseling materials, and lack of support from providers.^24^ This initiative was also studied in Tanzania, and a similar qualitative study revealed challenges with fidelity to the intervention and varied interpersonal aspects of care, with some women feeling rushed or pressured into using intrauterine contraceptive devices.^24^ Our research showed that the addition of the Medical Eligibility Criteria for Contraceptive use Mobile Application tool may relieve some of the stresses of needing a dedicated family planning counselor and counseling materials. Furthermore, while there were some quotes related to need for additional time for using the counseling intervention, many providers expressed that the intervention improved rapport and effectiveness of counseling which allowed them to feel their time was well spent. Finally, patients value respect from their providers, prioritization of their personal preferences and values, and individualized information exchange,^25^ which was described as occurring during the intervention.

### Implication for practice and research

Our findings have clinical and research implications for postpartum family planning care. Procedural and contextual adjustments, including incorporation of the strategy in antenatal care, and creation of private spaces on the postnatal ward for counseling, would enhance the effect of the strategy. Given the widespread use of mobile technology and the continued unmet need for postpartum contraception, a next step is to evaluate the implementation of the Postpartum Family Planning Package in a broader range of settings, including in lower-resource settings and among community health workers.

### Strengths and limitations

Strengths of our study included the strong basis in an implementation science framework, which we used to develop our study, create an interview guide, and analyze findings. The limitations of our research included the inclusion of relatively urban, well-resourced hospitals, which limits generalizability. In addition, it is very possible that participants provided overly positive responses to interview questions due to social desirability bias, as they were aware that the research team had developed and had an interest in the success of the intervention. Interviewers were research assistants who had not been involved in development of research materials, but it is possible that the participants associated the interviewers with the investigator team. Finally, providers generally had a positive cultural view of contraception in the postpartum period, which may also have positively influenced their responses.

## Conclusion

In conclusion, the Postpartum Family Planning Package was well received by providers, created trust, and improved information exchange between providers and clients, and was well supported by individual hospital leadership. Scaling our strategy to additional hospital systems and countries will be an essential next step in reducing the unmet need for postpartum contraception globally.

## CRediT authorship contribution statement

**Sarita Sonalkar:** Writing – original draft, Supervision, Project administration, Methodology, Formal analysis, Conceptualization. **Ernest Maya:** Writing – review & editing, Project administration, Methodology, Formal analysis, Conceptualization. **Chris Guure:** Writing – review & editing, Validation, Supervision, Methodology, Investigation, Formal analysis, Conceptualization. **Arden McAllister:** Writing – review & editing, Supervision, Project administration, Investigation, Formal analysis. **Dzifa Adimle Puplampu:** Writing – review & editing, Supervision, Project administration, Formal analysis. **Roseline Dansowaa Doe:** Writing – review & editing, Project administration, Methodology. **Mary Eluned Gaffield:** Writing – review & editing, Project administration, Methodology, Formal analysis, Conceptualization. **James Kiarie:** Writing – review & editing, Project administration, Investigation, Conceptualization.

## Appendix Supplementary materials

Image, application 1

## Footnotes

Supplementary material associated with this article can be found in the online version at doi:10.1016/j.xagr.2025.100506.
