# Supplementary material for: Providers' perceptions of implementing standardized postpartum family planning: a qualitative study of midwives and nurses in Ghana
Source: AJOG Glob Rep. 2025 May 12;5(2):100506. doi: 10.1016/j.xagr.2025.100506 (PMC12173112; doi:10.1016/j.xagr.2025.100506)
Supplement: Supplementary file 1 [file mmc1.docx]

PPFP Stepped-Wedge – In Depth Interview Guide (Providers)

In this guide, the PPFP training package refers to a package of these elements:

- 1. Education by experts on specific methods, side effects, benefits, and risks, as well as best practices in counseling.
- 2. Each postpartum nurse has and uses the WHO Medical Eligibility Criteria smartphone app during counseling
- 3. One-on-one family planning counseling for clients
- 4. Family planning nurses come to postpartum ward to support and supervise family planning counseling

We will be discussing each of these elements separately during the interview.

Part 1: Introduction

| **Overall feasibility, acceptability, appropriateness** | Tell me briefly about your experiences participating in the postpartum family planning study and the PPFP package.  -*What parts of it did you like? What parts did you dislike?* |
| --- | --- |

Part 2: Experiences with in-person training in postpartum family planning

For this section of the interview, please recall the in-person group training session that you participated in on [*date, specific to hospital*]. To remind you, the **content** of the training included family planning counseling, family planning methods, introduction to the MEC app, and cases that allowed practice in postpartum family planning and use of the app. The **methods** of training were three lectures, followed by interactive case studies.

| **Feasibility, acceptability, appropriateness** | -What was your experience with the training program?  *What did you like? What did you dislike?* |
| --- | --- |
|  | -How appropriate was the length and timing of training?  Probing questions  -*How did the length and timing of the training affect your engagement/attention?*  *-How did the length and timing of training affect your ability to remember/retain the information?*  *-What changes in duration, timing, or frequency of training would be helpful? [for example, longer session, shorter session, more sessions, different time of day]* |
|  | -How appropriate was the content of the training? Which content was most helpful? Which content was less helpful?  -How did the content of the training relate to your usual care and workflows? |
|  | -How appropriate were the methods of the training session?  -How did the methods of the training session affect your ability to remember/retain the information? |
| **Intervention characteristics** | |
| Evidence strength & quality | What do supervisors, directors of nursing, or other colleagues at your hospital think about the training session? |
| Relative Advantage | How did the training session compare to other methods of learning about postpartum family planning that you have experienced? |
| Adaptability | What kind of changes or modifications do you think can be made to the PPFP training package to have it work more effectively in your setting?  What other settings might this training be useful for? |
| Design Quality and Packaging | What is your perception of the quality of the the training that took place at your hospital? |
| **Inner setting** | |
| Structural characteristics | How does the infrastructure of the hospital affect your ability to attend the training? [Infrastructure can mean the size, physical layout, and other characteristics of the hospital and staffing.] |
| Networks and communications | -How did participation in the training that day affect clinical workflows? |
| **Sustainability** | |
|  | How easy or hard would it be to have ongoing postpartum family planning trainings at your hospital? |

Part 3: For this section, please think about how you use the WHO MEC app while counseling clients on the postpartum ward.

| **Feasibility, acceptability, appropriateness** | -What was your experience using the app while counseling clients? Probing questions  What did you like? What did you dislike? |
| --- | --- |
|  | Do you feel that it is acceptable and/or appropriate to use the MEC app while counseling?  Probing questions  Tell me about how use of the app impacted your workflow (or the workflow of those that you supervised).  Tell me about how use of the app impacted your clients (or the clients of those that you supervise). |
| **Intervention characteristics** | |
| Evidence strength & quality | What do supervisors, directors of nursing, and other colleagues at your hospital think about the WHO MEC app? |
| Relative Advantage | How does using the app for one-on-one counseling compare to how you were used to counseling clients?  Probing questions  What advantages are there to using the app, compared to other methods of recommending methods to clients? What disadvantages are there to using the app, compared to other methods of recommending methods to clients? |
| Adaptablity | What changes or modifications to the MEC app do you think can be made to make it more effective in your setting?  In what other settings might the app be useful? |
| Design Quality and Packaging | What is your perception of the quality of the app? |
| **Inner setting** | |
| Structural characteristics | How does the infrastructure of the hospital affect your ability to use the app? [Infrastructure can mean the size, physical layout, and other characteristics of the hospital and staffing.] |
| Networks and communications | How did working with other people affect how you used the app? |
| Culture | How did the hospital culture, workflows, and client numbers affect how you used the MEC app? |
| Implementation climate | How does using the counselling app align with other goals of your hospital?  What would help providers continue to be motivated to use the app while counseling clients? |
| **Sustainability** | |
|  | How sustainable is the use of the MEC app while counseling postpartum clients on the ward? |

Part 4: one-on-one counseling

| **Feasibility, acceptability, appropriateness** | -What was your experience with one-on-one counseling?  Probing questions  What did you like? What did you dislike?  *-What was your experience using the app while counseling? What did you like? What did you dislike?* |
| --- | --- |
|  | Tell me about the feasibility of this method of counseling. |
|  | Do you feel that it is acceptable and/or appropriate to do one-on-one counseling? |
|  | Tell me about how one-on-one counseling impacted your workflow (or the workflow of those that you supervised). |
|  | Tell me about how one-on-one counseling impacted your clients (or the clients of those that you supervise). |
|  | We found that client were choosing methods during one-on-one counseling but often not obtain the method before discharge. What is your perception of why this was happening? |
|  |  |
| **Intervention characteristics** | |
| Evidence strength & quality | What do the influential people at your hospital think about one-on-one counseling? |
| Relative Advantage | How does one-on-one counseling compare to how you were used to counseling clients?  What other methods of counselling would you recommend? |
| Adaptablity | What changes or modifications to one-on-one counseling would you recommend?  What kind of changes or modifications do you think can be made to the one-on-one counseling to have it work more effectively in your setting? |
| **Inner setting** | |
| Structural characteristics | How does the infrastructure of the hospital affect your ability to do one-on-one counseling? [Infrastructure can mean the size, physical layout, and other characteristics of the hospital and staffing.] |
|  | How does the infrastructure of the hospital affect your ability to give patients the methods they choose? [Infrastructure can mean the size, physical layout, and other characteristics of the hospital and staffing.] |
| Networks and communications | How did working with other people/staff affect your workflows doing one-on-one counseling? |
| Culture | How did the hospital setting (culture) affect whether and how you did one-on-one counseling?  Probing questions  How did the hospital setting (culture) help you with one-on-one counseling?  How did the hospital setting (culture) make it more difficult to do one-on-one counseling? |
| Implementation climate | How does doing one-on-one counseling align with other goals of your hospital?  What do you think we could do to help ensure that providers continue doing one-on-one counseling? |
| **Sustainability** | |
|  | How sustainable is doing one-on-one counseling in your facility?  Probing question  What would allow one-on-one counseling to be sustained at your hospital? |
|  | How would dedicated family planning personnel for counseling affect the ability continue one-on-one counseling*?* |
|  | What would allow you and your staff to offer placement of implants on the postpartum or gynecology ward before clients are discharged home?  What will make this practice hard?  What will make this practice rewarding? |
|  | What would allow you and your staff to offer injectables on the postpartum or gynecology ward before clients are discharged home?  What will make this practice hard?  What will make this practice rewarding? |
|  | What would allow you and your staff to offer progestin-only pills on the postpartum or gynecology ward before clients are discharged home?  What will make this practice hard?  What will make this practice rewarding? |

**Conclusions**

What additional thoughts do you have about the Postpartum Family Planning Package?

Thank you for your participation and your thoughts!

References

NCI White Paper on Qualitative Methodology in IS: <https://cancercontrol.cancer.gov/sites/default/files/2020-09/nci-dccps-implementationscience-whitepaper.pdf>

Qualitative methods in implementation research: an introduction:

<https://www.ncbi.nlm.nih.gov/pmc/articles/PMC7023962/>

cfirguide.org
